# The Characterization of Biological Rhythms in Mild Cognitive Impairment

**DOI:** 10.1155/2014/524971

**Published:** 2014-07-17

**Authors:** Elisabet Ortiz-Tudela, Antonio Martinez-Nicolas, Carmen Díaz-Mardomingo, Sara García-Herranz, Inmaculada Pereda-Pérez, Azucena Valencia, Herminia Peraita, César Venero, Juan Antonio Madrid, Maria Angeles Rol

**Affiliations:** ^1^Chronobiology Laboratory, Department of Physiology, Faculty of Biology, University of Murcia, Campus de Espinardo, Espinardo, 30100 Murcia, Spain; ^2^Department of Basic Psychology, Universidad Nacional de Educación a Distancia (UNED), 28040 Madrid, Spain; ^3^Department of Psychobiology, Universidad Nacional de Educación a Distancia (UNED), 28040 Madrid, Spain

## Abstract

*Introduction*. Patients with dementia, especially Alzheimer's disease, present several circadian impairments related to an accelerated perturbation of their biological clock that is caused by the illness itself and not merely age-related. Thus, the objective of this work was to elucidate whether these circadian system alterations were already present in patients with mild cognitive impairment (MCI), as compared to healthy age-matched subjects. *Methods*. 40 subjects (21 patients diagnosed with MCI, 74.1 ± 1.5 y.o., and 19 healthy subjects, 71.7 ± 1.4 y.o.) were subjected to ambulatory monitoring, recording wrist skin temperature, motor activity, body position, and the integrated variable TAP (including temperature, activity, and position) for one week. Nonparametrical analyses were then applied. *Results*. MCI patients exhibited a significant phase advance with respect to the healthy group for the following phase markers: temperature M5 (mean ± SEM: 04:20 ± 00:21 versus 02:52 ± 00:21) and L10 (14:35 ± 00:27 versus 13:24 ± 00:16) and TAP L5 (04:18 ± 00:14 versus 02:55 ± 00:30) and M10 (14:30 ± 00:18 versus 13:28 ± 00:23). *Conclusions*. These results suggest that significant advances in the biological clock begin to occur in MCI patients, evidenced by an accelerated aging of the circadian clock, as compared to a healthy population of the same age.

## 1. Introduction

Normal aging is accompanied by a progressive impairment of circadian rhythms [1]. These changes include higher fragmentation, lower amplitude, and a phase advance of the rest-activity and temperature rhythms [2–4] parallel to impairment of the sleep-wake architecture [2, 5]. The modifications observed with aging have also been related to a progressive impairment in cognitive performance [5]. Furthermore, an exacerbation of these changes in the circadian system is also found with Alzheimer's disease (AD) [6].

Mild cognitive impairment (MCI) is a heterogeneous condition in which subjects belonging to the amnestic subgroup present a high risk of developing dementia [7], with conversion rates to AD close to 12% per year [8]. Compared to the relatively large number of articles devoted to the study of circadian alterations in AD, only scarce and sometimes contradictory data are available on the possible impairment of the circadian system in MCI. In this sense, a phase delay of the rest-activity rhythm has been recognized as a risk factor for developing MCI [9, 10]. On the contrary, a phase advance in the dim light melatonin onset, the gold standard for circadian phase assessment, has been reported in MCI patients [11]. The use of a single circadian marker for circadian assessment in such patients with low circadian robustness could explain such discrepancies. Furthermore, internal desynchrony among the different rhythms could also account for these inconsistencies. As a result, reliable evaluation through multichannel ambulatory monitoring of the status of the circadian system is beginning to receive attention [12, 13]. A better understanding of the changes in this system would aid in the implementation of prevention therapies, such as controlled bright light therapy and/or melatonin administration, which have already proven their relevance in these pathologies [14].

To this end, in this study we propose the integration of 3 circadian rhythms, wrist temperature, motor activity, and body position, into the composite variable TAP [12] in order to describe circadian system alterations associated with MCI.

## 2. Methods

### 2.1. Study Group

Our study is part of a larger ongoing study in which 247 participants are being followed for several years in order to determine the prevalence and stability of the different MCI subtypes [15, 16]. The participants were recruited in the Autonomous Community of Madrid (ACM, Spain). The larger sample of participants was originally a mixed sample of individuals presenting mild cognitive impairment and unimpaired subjects.

The inclusion criteria resulted in a sample of subjects aged between 65 and 90 y.o. who volunteered to participate in the study and agreed to follow-up visits and different assessments. Conversely, medical conditions such as neurodegenerative disease, severe cognitive impairment, disabling chronic diseases, psychiatric disorders, marked neurological abnormalities (aphasia, agraphia, and/or apraxia), severe sensory deficits, depression, diabetes, and cerebrovascular accidents were considered to be exclusion criteria. Both the inclusion and exclusion criteria have been previously defined by Venero et al. (2013) [15].

From the initial group of 247 subjects participating in the previous study [15], we selected a subsample to match cognitive condition (based on psychometric criteria, according to the standard guidelines [17]), gender, and age between the mild cognitive impairment and control groups and who agreed to rhythm monitoring during a one-week period. Our subsample consisted of 40 subjects, 21 of whom were diagnosed as suffering from MCI (6 nonamnestic, 2 amnestic, and 13 multidomain) (74.1 ± 1.5 y.o.) and an age-matched healthy population of 19 subjects (71.7 ± 1.4 y.o., *P* > 0.05) for age comparisons between healthy and MCI subjects. The multidomain subgroup exhibited no differences in age with respect to the healthy group (71.7 ± 1.4 y.o. versus 73.9 ± 1.1 for healthy and MD subjects, respectively; *P* = 0.22). With respect to gender, the control group consisted of a sample of 14 women and 5 men, while the MCI group included 15 women and 6 men.

During this time, all subjects were submitted to cognitive assessment (including the episodic memory Verbal Learning Test from Complutense University, TAVEC; the Spanish version of the Mini-Mental State Examination (MMSE); the Yesavage Scale to assess the participant's emotional state; the Blessed Dementia Scale as a personal autonomy scale; several language tests for phonetic and semantic fluency; Trail Making Test-A measuring attention span; Trail Making Test-B and alternating graphs and loops for executive function; and the Rey-Osterrieth complex figure test for constructive praxis, among others), as detailed in Venero et al. (2013) [15]. These subjects also wore temperature, activity, body position, and luxometer sensors.

The study was approved by the Ethics and Research Committee at the UNED (Spanish National University of Distance Education) and the University of Murcia (SPAIN), and all participants provided written consent to perform the neuropsychological test battery and wear the sensors for one week. The study also abided by the Helsinki Declaration of 1975 (revised in 2013).

### 2.2. Rhythm Assessment

#### 2.2.1. Temperature Rhythm

Wrist temperature rhythm was assessed continuously for 7 days using a temperature sensor (Thermochron iButton DS1921H, Dallas, Maxim) programmed to sample once every 10 minutes; said monitoring device was attached as previously described [12, 18].

#### 2.2.2. Body Position and Rest-Activity Rhythm

The body position and rest-activity rhythms were assessed over the course of the same 7 days using an actimeter (Hobo Pendant G Acceleration Data Logger) placed on the nondominant arm.

The sensor was programmed to record data every 30 seconds, and the variables were obtained as described by Ortiz-Tudela et al. (2010) [12].

#### 2.2.3. TAP Computation

Wrist temperature, motor activity, and body position were combined according to the algorithms described by Ortiz-Tudela et al. (2010) [12] in order to obtain the integrated TAP variable. TAP values ranged from 0 to 1. Values near 1 indicate a low wrist temperature, a high level of activity, and a vertical arm position, suggesting a high level of activation, whereas values near 0 correspond to a high wrist temperature, a low level of activity, and a horizontal arm position, which is compatible with quiet sleep.

#### 2.2.4. Environmental Light-Exposure Recording

All subjects were required to wear a HOBO Pendant Temperature/Light Data Logger UA-002-64 (Onset Computer, Bourne, Massachusetts, USA) on a lanyard close to their eyes to record light exposure.

Participants were instructed to wear the luxometer over their clothing and to leave it on the bedside table as they slept, according to previously described methodology [19].

#### 2.2.5. Temporal Series Analysis

In order to characterize the circadian pattern for TAP and each individual variable, we performed the following nonparametric analysis (as previously described by van Someren et al. (1999) [20]).

*Interdaily Stability (IS).* This quantifies rhythm stability over different days. It varies between 0 for Gaussian noise and 1 for a perfect stability, where the rhythm repeats itself exactly, day after day.

*Intradaily Variability (IV).* This parameter shows the fragmentation of the rhythm. Its values oscillate between 0 (when the wave is perfectly sinusoidal) and 2 (Gaussian noise). 

*Phase Markers.* The timing of the ten or five consecutive hours with the lowest values (L10 and L5, resp.) and the timing of the ten or five consecutive hours with the highest values (M10 and M5, resp.) were calculated. M5 for temperature and L5 for activity, position, TAP, and light exposure represented the phase markers occurring during the night, while L10 for temperature and M10 for activity, position, TAP, and light exposure were the phase markers during the day. 

*Relative Amplitude (RA).* This refers to the difference between the mean values of the ten consecutive hours with the highest values and the mean values of the five consecutive hours with the lowest values (VM10 and VL5, resp.) divided by VM10 + VL5; to facilitate comparisons among variables, the difference between the VL10 and VM5 divided by VL10 + VM5 was also used. This parameter was multiplied by ten for temperature. 

*Circadian Function Index (CFI).* CFI incorporates three parameters (IV, IS, and RA) and can be calculated for any variable. IV values were inverted and normalized between 0 and 1, with 0 being a noise signal and 1 a perfect sinusoid. Finally, CFI was calculated as the average of these three parameters. Consequently, CFI oscillates between 0 (absence of circadian rhythmicity) and 1 (a robust circadian rhythm) [12]. 

*Wrist Temperature Increase Onset (WTiO).* This parameter has recently been described by Bonmati-Carrion et al. (2014) [21] and has been shown to be very closely correlated with the Dim Light Melatonin Onset (DLMO). According to its definition, we calculated L2 and M5 for wrist temperature and their corresponding VL2 and VM5 (i.e., the center of the period of the 2 and 5 consecutive hours of lowest (L2) and highest (M5) values of wrist temperature). Therefore, the WTiO corresponds to the time when 35% of the difference between L2 and M5 is achieved after L2.

### 2.3. Statistical Analyses

Firstly, descriptive statistics were calculated for every parameter chosen. Comparisons between the distribution of the recorded variables according to time (when averaging data on an hourly basis) and group (MCI or healthy) were performed by a repeated-measures ANOVA (significance level set at *P* < 0.05) and* post hoc* comparisons using Bonferroni's test.

A Student's *t*-test was applied when comparisons for demographic characteristics (age and cognitive state), nonparametrical parameters, and WTiO between two groups were performed. Thus, the values for each parameter were compared between healthy and MCI subjects and between healthy and multidomain subjects. The interest in evaluating the differences between the healthy and MD subgroups lies in the fact that the multidomain subgroup exhibits a more severe cognitive impairment and a brain activity similar to that seen in Alzheimer's disease patients (López et al., 2014) [22].

The statistical analyses were performed using the PASW Statistics 18 software (IBM, USA).

## 3. Results

### 3.1. Overall Rhythm View

Both MCI patients and healthy subjects showed altered circadian patterns of wrist temperature, motor activity, body position, and TAP that were characteristic of aged persons [1, 3, 4, 12, 19, 21, 23, 24]. However, subjects with MCI showed a clear phase advance for some markers of the circadian system.

**Figure 1:**
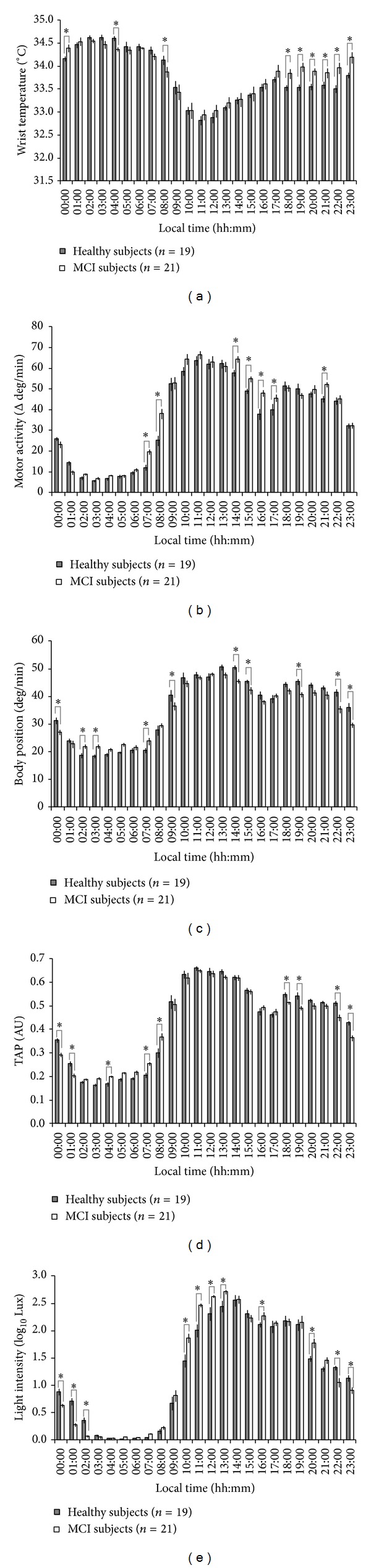
Rhythms of all variables studied in control and mild cognitively impaired subjects (MCI). Hourly values were first averaged per day and then per consecutive days in order to obtain these mean waveforms. Wrist temperature, motor activity, body position, TAP, and light exposure mean waveforms are shown in panels (a), (b), (c), (d), and (e), respectively. Grey bars correspond to healthy individuals and white bars to the MCI group. Values are expressed as mean ± SEM. Statistically significant differences obtained after repeated-measures ANOVA +* post hoc* Bonferroni between the two groups studied for each time point are marked with an asterisk.

With respect to the wrist temperature rhythm (Figure 1(a)), the wake maintenance zone disappeared in both groups. This time interval of minimal sleep probability coincides with minimal values of daily distal temperatures in healthy adults, usually between 20:00 and 22:00 h [12, 18]. This is in contrast to our findings, where the minimal values were shifted to just after the moment of awakening. However, this alteration was more pronounced in MCI subjects, who presented statistically significant higher temperature values (*P* < 0.05) than healthy controls between 20:00 h and 22:00 h but also at 17:00 h, 18:00 h, 23:00 h, and 00:00 h.

The motor activity rhythm (Figure 1(b)) exhibited significantly higher values (*P* < 0.05) for MCI subjects at 07:00 h and 08:00 h (suggesting an earlier activation of these subjects), around the postprandial hours (14:00 to 17:00), and at 21:00 h with respect to healthy subjects.

Both body position (Figure 1(c)) and the integrated variable TAP (Figure 1(d)) exhibited a general trend towards nocturnal activation in MCI subjects. Statistically significant (*P* < 0.05) higher values were found in the MCI group for TAP at 04:00 h and 07:00 h and for body position at 02:00 h, 03:00 h, and 07:00 h. However, a reduced activation was detected during the daytime in MCI, with lower statistically significant values for comparisons between MCI and healthy subjects for TAP at 18:00 h, 19:00 h, 22:00 h, and 23:00 h and for body position at 14:00 h, 15:00 h, 19:00 h, 22:00 h, and 23:00 h, *P* < 0.05.

MCI subjects were exposed to high levels of light (Figure 1(e)) earlier than healthy subjects, as their light intensity levels were significantly higher from 10:00 h to 13:00 h, and at 16:00 h and 20:00 h. On the contrary, healthy subjects showed significantly higher light exposure from 22:00 h to 02:00 h.

### 3.2. Nonparametrical Characterization of Rhythms

When comparing nonparametrical indexes between healthy and MCI groups, significant differences were found with respect to timing (Table 1). Thus, a consistent phase advance in MCI subjects was evident for wrist temperature M5 and L10, as well as for TAP L5 and M10.

Furthermore, when focusing on the most severely affected MCI subjects in our sample as determined by lower scores on the Mini-Mental State Examination and the psychometric evaluation [15], that is, the multidomain (MD) subgroup (*n* = 13), a phase advance was once again confirmed for wrist temperature rhythm M5 (*P* = 0.009) and L10 (*P* = 0.042) and for TAP L5 (*P* = 0.012), as compared to healthy subjects (Table 1). Although this phase advance was consistently observed for the remaining variables, statistical significance was not achieved. When examining the multidomain group (*n* = 13) with respect to the remaining mild cognitive impaired subjects (*n* = 8), that is, MCI subjects not included in the MD subgroup, we found a significant phase advance in the MD subgroup for M10 (15:29 ± 00:46 versus 13:31 ± 00:21, *P* = 0.02), a phase marker for the rest-activity rhythm.

The WTiO parameter was determined to occur at 22:48 ± 00:34 h and 21:49 ± 00:23 h for healthy and mild cognitively impaired patients, respectively. Despite the tendency to be phase-advanced in MCI subjects as well, this difference was not significant (*P* = 0.156).

## 4. Discussion

Our study, complementing that previously published by Venero et al. (2013) [15], highlights differences that begin to occur in the circadian timing system of MCI subjects. We found that the MCI group presented a phase advance in temperature, activity, position, TAP, and light exposure rhythms with respect to a healthy age-matched population.

Inconsistent changes in circadian timing have already been reported for the rest-activity rhythm in MCI. In this sense, Cochrane et al. (2012) [25] observed a phase delay in this rhythm in MCI subjects. Furthermore, other studies have identified this phase delay of the rest-activity rhythm as a predictor for developing MCI [9, 10]. However, a recent study combining polysomnography, actigraphy, and melatonin analysis showed a clear phase advance in sleep and dim light melatonin onset in patients with MCI [11], similar to that observed by us using rhythmic multivariable recordings. Moreover, in our study, this phase advance was also significant in the subgroup of patients with MD, characterized by multiple domain impairment, in contrast to single amnestic MCI and nonamnestic MCI. This circadian alteration is similar to that observed during normal aging, since elderly people consistently show a phase advance in their circadian rhythms [2]. This discrepancy between our study and the results of Cochrane (2012) [25], Schlosser Covell et al. (2012) [10], and Tranah (2011) [9] could be possibly attributed to how phases are calculated. These authors calculate the acrophase of the rest-activity rhythm using the cosinor method. Thus, a sine wave fit to a time series [26] is compulsory, while a nonparametrical rhythm characterization does not assume any waveform a priori [20]. The rest-activity rhythm resembles more a squared wave than a sine wave, and given the nocturnal agitation called “sundowning” that appears in AD [6, 8], the cosinor may delay the moment of maximum activity (acrophase).

## 5. Conclusions

Altogether, and taking into account the exploratory nature of the study, it seems that MCI subjects begin to experience some of the early circadian rhythms disturbances associated with premature aging and AD, which are especially patent in those subjects categorized in the multidomain group. Furthermore, the TAP variable may be a relevant tool for these studies, in which a multiple-rhythm assessment could contribute to a better understanding of the pathology studied. This is especially true considering that TAP provides a more global insight into circadian system status, overcoming artifacts that influence isolated variables. These results emphasize the potential benefits of chronoenhancement therapies already proposed [8], which could help resynchronize the biological clock and thus prevent some of the symptoms suffered by these patients.

## Figures and Tables

**Table 1:** Nonparametrical characterization of the study group.

	Healthy	MCI	MD	MCI except MD
Wrist temperature				
IS	0.49 ± 0.04	0.43 ± 0.04	0.41 ± 0.05	0.46 ± 0.07
IV	0.20 ± 0.02	0.20 ± 0.02	0.19 ± 0.03	0.22 ± 0.03
RA	0.25 ± 0.02	0.23 ± 0.02	0.22 ± 0.02	0.25 ± 0.05
M5	**04:20** ± **00:21** ^1a^	**02:52** ± **00:21** ^2^	**02:53** ± **00:22** ^b^	06:26 ± 02:31
L10	**14:35** ± **00:27** ^1a^	**13:24** ± **00:16** ^2^	**13:18** ± **00:20** ^b^	14:32 ± 00:50
CFI	0.55 ± 0.02	0.52 ± 0.02	0.51 ± 0.02	0.53 ± 0.04

Motor activity				
IS	0.45 ± 0.02	0.46 ± 0.02	0.49 ± 0.02	0.41 ± 0.05
IV	0.77 ± 0.03	0.73 ± 0.03	0.73 ± 0.03	0.73 ± 0.05
RA	0.78 ± 0.02	0.80 ± 0.02	0.80 ± 0.03	0.81 ± 0.04
L5	03:48 ± 00:11	03:23 ± 00:13	03:10 ± 00:17	03:55 ± 00:25
M10	14:34 ± 00:17	14:08 ± 00:21	**13:31** ± 00:21*	**15:29** ± 00:46*
CFI	0.62 ± 0.02	0.63 ± 0.01	0.64 ± 0.02	0.62 ± 0.02

Body position				
IS	0.50 ± 0.04	0.47 ± 0.05	0.49 ± 0.06	0.44 ± 0.07
IV	0.31 ± 0.02	0.33 ± 0.03	0.32 ± 0.03	0.34 ± 0.05
RA	0.47 ± 0.04	0.43 ± 0.04	0.45 ± 0.05	0.41 ± 0.06
L5	04:34 ± 00:29	02:53± 00:47	03:01 ± 00:57	03:46 ± 01:21
M10	14:30 ± 00:45	12:57 ± 00:57	12:50 ± 01:17	12:01 ± 01:37
CFI	0.61 ± 0.03	0.58 ± 0.03	0.59 ± 0.04	0.56 ± 0.04

TAP				
IS	0.61 ± 0.03	0.57 ± 0.04	0.60 ± 0.04	0.53 ± 0.10
IV	0.26 ± 0.02	0.28 ± 0.02	0.28 ± 0.02	0.27 ± 0.04
RA	0.55 ± 0.03	0.49 ± 0.04	0.51 ± 0.04	0.48 ± 0.08
L5	**04:18** ± **00:14** ^1a^	**02:55** ± **00:30** ^2^	**03:15** ± **00:19** ^b^	03:36 ± 00:27
M10	**14:30 ** ± **00:18** ^1a^	**13:28** ± **00:23** ^2^	13:30 ± 00:23	13:57 ± 00:54
CFI	0.68 ± 0.02	0.64 ± 0.03	0.65 ± 0.03	0.62 ± 0.06

Light exposure				
IS	0.64 ± 0.03	0.68 ± 0.02	0.67 ± 0.02	0.70 ± 0.03
IV	0.26 ± 0.02	0.25 ± 0.01	0.27 ± 0.01	0.23 ± 0.02
RA	0.98 ± 0.01	0.97 ± 0.01	0.98 ± 0.01	0.96 ± 0.02
L5	04:17 ± 00:09	03:56 ± 00:13	03:43 ± 00:08	04:17 ± 00:20
M10	14:29 ± 00:15	14:18 ± 00:05	14:11 ± 00:05	14:29 ± 00:07
CFI	0.83 ± 0.01	0.84 ± 0.01	0.84 ± 0.01	0.85 ± 0.02

These indexes were calculated throughout the study for the healthy group (*n* = 19), the cognitively impaired group (MCI, *n* = 21), the multidomain subgroup (MD, *n* = 13), and MCI patients excluding the multidomain subgroup (MCI except MD, *n* = 8).

Student's *t*-tests were used to compare the healthy and MCI groups (different numbers, “1” and “2”, indicate statistically significant differences), the healthy and MD subgroups (different letters, “a” and “b”, indicate statistically significant differences), and the MD subgroup and the rest of the MCI patients (an asterisk marks statistically significant differences). In addition, significant differences are highlighted in bold.

All values are expressed as mean ± SEM.

IS stands for interdaily stability; IV for intradaily variability; RA for relative amplitude; M5 and M10 for the center of the consecutive period of 5 and 10 hours of maximum values, respectively; L10 and L5 indicate the consecutive 10- and 5-hour periods of minimum values, respectively; and CFI corresponds to the circadian function index.
